# Participant characteristics associated with the effects of a physical and cognitive training program on executive functions

**DOI:** 10.3389/fnagi.2022.1038673

**Published:** 2022-10-25

**Authors:** Anna Tirkkonen, Timo Törmäkangas, Jenni Kulmala, Tuomo Hänninen, Anna Stigsdotter Neely, Sarianna Sipilä

**Affiliations:** ^1^Gerontology Research Center, Faculty of Sport and Health Sciences, University of Jyväskylä, Jyväskylä, Finland; ^2^Faculty of Social Sciences (Health Sciences) and Gerontology Research Centre, Tampere University, Tampere, Finland; ^3^Division of Clinical Geriatrics, Center for Alzheimer Research, Department of Neurobiology, Care Sciences, and Society (NVS), Karolinska Institutet, Stockholm, Sweden; ^4^Population Health Unit, Finnish Institute for Health and Welfare, Helsinki, Finland; ^5^NeuroCenter, Department of Neurology, Kuopio University Hospital, Kuopio, Finland; ^6^Department of Social and Psychological Studies, Karlstad University, Karlstad, Sweden; ^7^Engineering Psychology, Luleå University of Technology, Luleå, Sweden

**Keywords:** older adults, executive functions, training response, physical training, physical and cognitive training

## Abstract

**Background:**

Physical and cognitive interventions have been shown to induce positive effects on older adults’ executive functioning. However, since participants with different background characteristics may respond differently to such interventions, we investigated whether training effects on executive functions were associated with sex, training compliance, and age. We also investigated if change in global cognition was associated with physical and cognitive training intervention-induced changes in executive functions.

**Methods:**

Exploratory data from a randomized controlled trial were analyzed. Participants were 70–85-year-old men and women who received a 12-month physical (PT) or physical and cognitive training (PTCT) intervention. Measurements of executive functions related to inhibition (Stroop), set shifting (Trail Making Test B) and updating (Verbal Fluency) were performed at baseline and 12 months. Data were analyzed using a longitudinal linear path model for the two measurements occasion.

**Results:**

Stroop improved significantly more in women and participants in the low compliance subgroup who received PTCT than in counterparts in the PT subgroup (difference –8.758, *p* = 0.001 and difference –8.405, *p* = 0.010, respectively). In addition, TMT B improved after the intervention in the low compliance PTCT subgroup and worsened in the corresponding PT subgroup (difference –15.034, *p* = 0.032). No other significant associations were observed.

**Conclusion:**

Executive functions in women and in the participants, who only occasionally engaged in training showed greater improvement after the PTCT than PT intervention. However, the additional extra benefit gained from the PTCT intervention was uniquely expressed in each executive function measured in this study.

## Introduction

Executive functions are high-order cognitive functions that enables independent, appropriate and self-serving behavior (Harada et al., 2013). It is generally agreed that executive functions are consist of three sub-domains, inhibition, set shifting and updating that are united but show also diversity and serve as a base, for example, for problem solving, reasoning and planning (Miyake et al., 2000; Diamond, 2013). Executive functions has been shown to be prone age-related decline (Harada et al., 2013), however this decline can be attenuated with training (Diamond, 2013).

Physical and cognitive interventions have been shown to induce positive effects on older adults’ executive functioning (Ten Brinke et al., 2020; Sipilä et al., 2021; Han et al., 2022). However, participants with different baseline characteristics may respond differently to different training interventions. To develop optimized interventions and guidelines for executive functioning among older people, the factors that may influence training responses need to be identified. The results of PASSWORD, our earlier randomized controlled study (Sipilä et al., 2021), showed that a 12-month multicomponent physical training program combined with computer-based cognitive training improved executive functions related to inhibition more compared to physical training alone among older adults who did not meet physical activity guidelines at baseline. However, no significant intervention-induced changes between the study groups were observed in other domains of executive functions namely set shifting or updating.

Previous research findings suggest that the training response of physical and cognitive training (PTCT) interventions are depended on sex, training frequency and age, although of the previous studies are somewhat inconsistent. For example, meta-analysis by Barha et al. (2017) found that women executive functioning gained greater benefit from physical training interventions compared to men. However, recent randomized controlled trial (Roig-Coll et al., 2020) did not found sex differences in exercise efficacy after combined PTCT. Additionally, previous meta-analysis has suggested that high frequency of combined PTCT (5 times a week or more) is inefficient for executive functions (Zhu et al., 2016). Physical training, in turn, has been suggested to be most beneficial for executive functions when training frequency is rather frequent (3–5 times a week) in meta-analysis by Karr et al. (2014). Moreover, previous meta-analyses have been shown that, among older adults, older age was associated with greater positive intervention induced change in executive functions after combined PTCT (Zhu et al., 2016; Han et al., 2022). Concerning the physical training it might be that age does not have a similar role (Karr et al., 2014). Finally, better global cognition has been show to correlate with better performance in executive functions (Shao et al., 2020).

In this hypothesis-generating analysis, we investigated whether the training responses observed in different domains of executive functioning, inhibition, set shifting and updating were dependent on sex, training compliance or age. We further investigated if change in global cognition was associated with intervention-induced change in executive functions sub-domains.

## Methods

### Study design

This study utilized data from our earlier assessor-blinded randomized controlled trial.^1^ The study design and the results have been published previously (Sipilä et al., 2018, 2021). Ethical approval of the study was obtained from the Ethical Committee of Central Finland Health Care District (14/12/2016, ref: 11/2016). All participants gave a written consent before the baseline measurements.

### Participants

Participants were 70–85-year-old community-dwelling men and women living in Jyväskylä, Finland and were randomly extracted from Finland’s Population Information System administered by the Population Register Center. The inclusion criteria were willingness to participate, not meeting physical activity guidelines (less than 150 min of moderate activity/week and no regular resistance training), ability to walk 500 meters without assistance, and a Mini-Mental State Examination (MMSE) score ≥ 24. The exclusion criteria were a severe chronic condition and/or medication affecting cognitive and/or physical performance, any contraindication for walking for physical training or walking tests, depressive mood (GDS-15 > 5 points and not having the self-reported or physician and primary investigator-assessed resources to commit to the study), risk-level use of alcohol (> 7 units per week for women and 14 for men), or any other contraindications for physical training or another member of the household participating in the PASSWORD-study. After exclusions, 314 participants were recruited to the study.

### Randomization and blinding

Participants were randomized in a 1:1 ratio and stratified by age (70–74, 75–79, 80–85) and sex into the PTCT (*n* = 155) or Physical Training alone (PT) (*n* = 159) groups.

### Interventions

The interventions have been described previously (Sipilä et al., 2021). In brief, both groups received a 12-month multicomponent progressive physical training intervention which was designed on the basis of physical activity guidelines and earlier studies (Sihvonen et al., 2004; Portegijs et al., 2008; Fielding et al., 2011). The PT intervention included two supervised training sessions a week, one for resistance and balance exercises and the other for walking and dynamic balance exercises. Resistance and balance exercises included short warm-up, balance exercises and strengthening resistance exercises for lower limbs, trunk, and upper body. Walking and dynamic balance sessions included 10-min warm-up and dynamic balance training, following 10–20 min continuously walk with target intensity of somewhat hard to hard. Supervised resistance training session took place in senior gyms equipped with machines utilizing air pressure technology.^2^ Supervised walking and dynamic balance training sessions took place outdoors except during winter months indoor sports hall. In addition, participants received a progressive home exercise program which was instructed to perform 2–3 times a week. Home exercise program included strength exercises for lower limbs, balance exercises and stretching. The PTCT group received also 12-month progressive computer-based cognitive training intervention targeted at improving executive functions. Participants were instructed to perform cognitive training 3–4 times a week.

### Measurements

Executive functions were assessed at baseline, 6 and 12 months. Other outcomes were assessed at baseline and 12 months. This study utilized data from the baseline and 12 months measurements for all outcomes.

#### Executive functions

Inhibition was assessed with the Color-Word Stroop test. In this test participants were requested to name colors in incongruent and congruent conditions. Finally, the time difference between two conditions was calculated and used as the outcome (Graf et al., 1995). *Set shifting* was assessed with the Trail Making Test Part B. This test requires participants to alternately combine numbers and letters as quickly as possible (Reitan, 1958). *Updating* was assessed with the Verbal Fluency Test. This test requires participants to name as many words beginning with P, A or S as they can in three separate 1-min trials (Koivisto et al., 1992). The total score is the summed number of the named words.

#### Participant characteristics

Subgroup analyses on age, sex, global cognition, and training compliance were pre-specified for walking speed, the main outcome, of PASSWORD (Sipilä et al., 2021). Participant *sex* and *age* were drawn from Finland’s Population Information System administered by the Population Register Center. *Global cognition* was assessed at baseline and after the interventions with the Consortium to Establish a Registry for Alzheimer’s Disease (CERAD) (Chandler et al., 2005; Paajanen et al., 2010). Compliance was based on participation in supervised training sessions. The high-compliance PT subgroup participated in at least 50% and the low-compliance PT subgroup in less than 50% of the supervised PT sessions. The high-compliance PTCT subgroup participated in at least 50% of the supervised PT sessions and performed Cognitive training (CT) at least twice a week. The low compliance PTCT subgroup participated in less than 50% of the supervised PT sessions and/or performed CT less than twice a week. The mean compliance for each program (walking and dynamic balance sessions: 59% in the PT group and 62% in the PTCT group. Resistance and balance sessions: 72% in PT group and 77% in PTCT group. Cognitive training: on average 1.9 times a week) has been published previously (Sipilä et al., 2021).

#### Descriptive variables

Body *height* and *weight* were measured at baseline, and BMI was calculated. *Education, current physical activity, smoking status*, and *self-rated health* were self- reported. *Education* was categorized as low (primary school or less) medium (middle school, folk high school, vocational school, or secondary school) or high (high school or university). Current physical activity was assessed with a seven-point scale (Hirvensalo et al., 2000) and re-categorized as high (categories 5–7) medium (categories 3 and 4) or low (categories 1 and 2). S*moking status* was categorized as never smokers (never smoker/less than 100 times), former smokers (never smoked regularly but smoked over 100 times) or current smoker (current smoker, regularly or occasionally). *Self-rated health* was reported as very good/good or average/poor.

### Statistical analyses

As descriptive statics we report the means and standard deviations for continuous variables and frequencies and percentages for categorical variables separately for PT and PTCT groups.

To identify potential outliers for the outcome variables of the main analyses, the outcome distributions were inspected graphically using univariate histograms and quantile plots and bivariate scatter plots. Skew and kurtosis were considered as summary statistics of distribution shape. While the plots, skew, and kurtosis (absolute value less than unity) indicated acceptable shape of distribution for all variables.

For the analyses, the outcomes were regressed within measurement waves in a two-group linear path model accounting for longitudinal correlation in the outcome variables. Using custom contrasts, we computed the differences in the regression coefficients from the measurement waves as the effects of time, and the difference in these time effects between groups was computed as the interaction effect.

Outcomes were tested for group-interaction over time using an interaction contrast in a linear model for the two longitudinal measurements. The model structure was set similar to the linear mixed model in order to account for within-person correlation, but it also permitted more general outcome variance structure specification and flexible handling of missing data with the maximum likelihood approach based on the missing-at-random (MAR) assumption. For continuous exposure variables (age and Cerad) the model included a within-subject part for the repeated measurements for each subject, and a between-subjects part contrasting the PTCT and PT groups based on regression coefficient differences. A similar model was used for binary exposure variables (sex and training compliance), but now the contrast was based on differences in expected marginal means. We report group means for each available measurement wave and group-by-time interactions as primary significance tests. As further subgroup contrasts, we also compared if the background factors and descriptive variables differed between PTCT and PT groups among men and women and high and low compliance groups. This analysis was carried out in SPSS for windows, version 26. The specific contrasts used for all comparisons are shown in the model and contrast specification section of the supplementary document. The path model analyses were conducted in Mplus, version 7.4 (Muthen and Muthén, 2017).

## Results

Participants’ mean age was in PT group was 74.4 and in PTCT group 74.5 years, approximately 60% of participants were women in both groups, 25% in PT group and 18% in PTCT group had the high level of education (Table 1).

**TABLE 1:** Participant’s characteristics.

	PTCT (*n* = 155)	PT (*n* = 159)
Age (years)	74.4 ± 3.9	74.5 ± 3.8
BMI	28.0 ± 4.9	27.9 ± 4.5
MMSE	27.9 ± 1.4	27.4 ± 1.5
CERAD	79.5 ± 8	79.0 ± 8.2
**SEX, no (%)**		
Feman	96 (62)	92 (58)
Man	59 (38)	67 (42)
**Education, no (%)**		
Low	23 (15)	25 (16)
Medium	94 (61)	106 (67)
High	38 (25)	28 (18)
**Current physical activity, no (%)**		
Low	56 (36)	70 (44)
Medium	80 (52)	68 (43)
High	19 (12)	21 (13)
**Smoking status, no (%)**		
Never	94 (61)	97 (61)
Former	52 (34)	57 (36)
Current	9 (6)	5 (3)
**Self-rated health, no (%)**		
Very good/good	73 (47)	68 (43)
Average/poor	82 (53)	91 (57)

Means and standard deviations. Frequencies and percentages. PTCT, Physical and cognitive training; PT, Physical training; BMI, Body Mass Index; MMSE, Mini-Mental State Examination; CERAD, Consortium to Establish a Registry for Alzheimer’s Disease.

Our previous study, suggest that PTCT group improved significantly more their performance in Stroop than PT group (Sipilä et al., 2021). This subgroup analysis shows that women, and the low-compliance groups in PTCT improved Stroop performance significantly more than in the corresponding PT subgroups (difference -8.758, *p* = 0.001 and difference -8.405, *p* = 0.010, respectively) (Table 2). In men and the high-compliance subgroups, no significant differences between the PT and PTCT interventions were observed. Age or global cognition were not significantly associated with the interventions-induced changes in Stroop performance.

**TABLE 2:** Means with 95% confidence intervals and unstandardized regression coefficients (B) with 95% confidence intervals for subgroup analysis of the Stroop outcomes.

Stroop^a^			PTCT				PT			PTCT-PT			
				95% CI				95% CI			95% CI		
Subgroup	*N*		Mean	Lower	Upper	*N*	Mean	Lower	Upper	Difference	Lower	Upper	*P*-value
Overall	155	BL	45.1	41.9	48.4	159	48.1	43.7	52.6	–6.766	–11.111	–2.421	0.002
		FU	34.0	31.2	36.8		43.8	40.5	47.1				
Men	59	BL	42.5	38.0	47.1	67	50.8	42.5	59.2	–3.575	–10.942	3.793	0.342
		FU	34.6	29.8	39.4		46.5	40.8	52.2				
Women	96	BL	46.7	42.3	51.2	95	46.2	41.6	50.8	–8.758	–13.873	–3.643	0.001
		FU	33.6	30.2	37.0		41.8	38.0	45.7				
Low compliance	95	BL	46.2	42.1	50.2	55	48.2	42.5	53.9	–8.405	–14.780	–2.031	0.010
		FU	35.1	31.5	38.7		45.5	39.9	51.1				
High compliance	60	BL	43.5	38.1	48.9	103	48.3	42.2	54.4	–5.966	–12.736	0.804	0.084
		FU	32.3	28.0	36.6		43.0	39.0	47.1				

			**B**	**Lower**	**Upper**		**B**	**Lower**	**Upper**	**Difference**	**Lower**	**Upper**	***P*-value**
	
Age	155	BL	0.860	0.026	1.695	159	1.037	–0.126	2.200	0.654	–0.482	1.790	0.259
		FU	1.368	0.686	2.049		0.890	0.032	1.749				
CERAD^b^	141	BL	–0.538	–0.939	–0.136	147	–0.691	–1.141	–0.241	–0.090	–0.655	0.474	0.754
		FU	–0.514	–0.810	–0.218		–0.577	–0.902	–0.252				

^a^Lower time indicates better performance, ^b^Comprises verbal fluency, modified Boston naming test, word lists constructional praxis, word list recall and word list recognition discriminability (total sum score range 0–100). B, unstandardized regression coefficient; CI, confidence interval; PTCT, physical and cognitive training; PT, physical training; CERAD, Consortium to Establish a Registry for Alzheimer’s Disease. Men vs. Women, *p* = 0.257, Training less vs. more than 50% (PT only), *p* = 0.596, Training less vs. more than 50% (PT and CT), *p* = 0.607.

Post-intervention TMT B performance improved significantly in the low compliance PTCT subgroup and worsened, i.e., was below the baseline level, in the low compliance PT subgroup (difference -15.034, *p* = 0.032) (Table 3). No significant differences in the change in TMT B change were observed among men, women, or the high-compliance subgroups. Moreover, no significant associations of age or global cognition with change in TMT B by intervention type were observed.

**TABLE 3:** Means with 95% confidence intervals and unstandardized regression coefficients (B) with 95% confidence intervals for subgroup analysis of the TMT-B outcomes.

TMT-B^a^			PTCT				PT			PTCT-PT			
				95% CI				95% CI			95% CI		
Subgroup		*N*	Mean	Lower	Upper	*N*	Mean	Lower	Upper	Difference	Lower	Upper	*P*-value
Overall	BL	155	130.7	120.8	140.6	158	132.2	123.7	140.8	–5.349	–13.620	2.922	0.205
	FU		119.3	108.6	129.9		126.1	117.7	134.6				
Men	BL	59	134.4	120.3	148.4	67	143.1	127.4	158.7	–13.486	–27.924	0.952	0.067
	FU		117.2	104.7	129.7		139.4	123.0	155.8				
Women	BL	96	128.5	115.1	141.9	91	124.3	115.3	133.3	–0.494	–10.190	9.202	0.920
	FU		119.9	104.8	135.0		116.2	108.6	123.7				
Low compliance	BL	95	137.4	123.2	151.6	54	125.8	112.8	138.7	–15.034	–28.769	–1.299	0.032
	FU		127.9	112.0	143.8		131.3	115.2	147.4				
High compliance	BL	60	120.2	108.6	131.8	103	135.9	124.8	147.1	–1.775	–13.150	9.599	0.760
	FU		106.7	95.5	117.8		124.2	114.3	134.1				

			**B**	**Lower**	**Upper**		**B**	**Lower**	**Upper**	**Difference**	**Lower**	**Upper**	***P*-value**
	
Age	BL	155	5.358	2.941	7.774	158	3.741	1.537	5.945	–1.574	–3.747	0.599	0.156
	FU		4.470	1.802	7.138		4.427	2.276	6.578				
CERAD^b^	BL	141	–1.317	–2.148	–0.486	147	–2.939	–3.788	–2.089	0.268	–0.826	1.362	0.631
	FU		–1.136	–1.968	–0.303		–3.026	–3.830	–2.222				

^a^Lower time indicates better performance, ^b^Comprises of verbal fluency, modified Boston naming test, word lists, constructional praxis, word list recall and word list recognition discriminability (total sum score range 0–100). B, unstandardized regression coefficient; CI, confidence interval; PTCT, physical and cognitive training; PT, physical training; CERAD, Consortium to Establish a Registry for Alzheimer’s Disease. Men vs. Women, *p* = 0.143, Training less vs. more than 50% (PT only), *p* = 0.001, Training less vs. more than 50% (PT and CT), *p* = 0.145.

No significant differences in change in Verbal Fluency were observed between the study subgroups (Table 4) and none of the participant characteristics were significantly associated with change in Verbal Fluency by intervention type.

**TABLE 4:** Means with 95% confidence intervals and unstandardized regression coefficients (B) with 95% confidence intervals for subgroup analysis of the verbal fluency outcomes.

Verbal fluency^a^			PTCT				PT			PTCT-PT			
				95% CI				95% CI			95% CI		
Subgroup		*N*	Mean	Lower	Upper	*N*	Mean	Lower	Upper	Difference	Lower	Upper	*P*-value
Overall	BL	155	42.3	40.3	44.4	159	40.9	38.9	42.9	0.291	–1.753	2.335	0.780
	FU		46.0	43.7	48.3		44.3	42.2	46.4				
Men	BL	59	39.5	36.0	43.0	67	35.7	32.7	38.7	–0.937	–4.116	2.241	0.563
	FU		41.4	37.6	45.1		38.6	35.4	41.7				
Women	BL	96	44.1	41.6	46.6	92	44.7	42.3	47.1	0.894	–1.725	3.514	0.503
	FU		48.8	46.1	51.5		48.5	46.0	50.9				
Low compliance	BL	95	41.2	38.8	43.9	55	40.9	37.8	44.0	–0.436	–3.551	2.676	0.784
	FU		44.2	41.4	47.0		44.3	40.9	47.7				
High compliance	BL	60	44.1	40.9	47.3	103	40.9	38.2	43.5	1.424	–1.284	4.133	0.303
	FU		48.9	45.1	52.7		44.2	41.6	46.9				

			**B**	**Lower**	**Upper**		**B**	**Lower**	**Upper**	**Difference**	**Lower**	**Upper**	***P*-value**
	
Age	BL	155	0.141	–0.392	0.675	159	–0.449	–0.975	0.078	–0.173	–0.705	0.360	0.525
	FU		–0.099	–0.689	0.492		–0.516	–1.066	0.035				
CERAD^b^	BL	141	0.539	0.328	0.749	147	0.409	0.207	0.611	–0.075	–0.350	0.200	0.593
	FU		0.510	0.296	0.724		0.455	0.252	0.659				

^a^Higher number of named words indicates better performance. ^b^Comprises of verbal fluency, modified Boston naming test, word lists, constructional praxis, word list recall and word list recognition discriminability (total sum score range 0–100). B, unstandardized regression coefficient; CI, confidence interval; PTCT, physical and cognitive training; PT, physical training. Men vs. Women, *p* = 0.383, *p* = 0.863, Training less vs. more than 50% (PT and CT), *p* = 0.377.

## Discussion

In this hypothesis-generating analysis, we investigated whether the training responses of different sub-domains of executive functioning were dependent on age, sex, training compliance or change in global cognition. Our earlier study suggested that the 1-year PTCT intervention provided additional benefit for inhibition compared to physical training alone (Sipilä et al., 2021). The present subgroup analyses suggests that the benefit found for inhibition may be driven by women sex and/or low compliance, defined as participation in less than half of the training. However, we found no statistically significant interactions between the sex subgroups and high- and low-compliance subgroups. The set shifting results showed that the participants in the low-compliance subgroup benefitted more from the PTCT than from physical training alone.

We found that women who received the PTCT showed a significantly greater improvement in their post-intervention inhibition performance than those who received physical training alone. Women who received PTCT improved their performance in Stroop by 28% whereas women who received physical training alone improved their performance by 9%. The corresponding changes among men indicated a similar but lower, statistically non-significant effect. Men receiving PTCT improved their performance in Stroop by 19% and men receiving physical training by 9%. The mechanism underlying the lower mean response rate among men remains unclear. It may be that activation of the neural circuits and/or molecular mechanisms (Grissom and Reyes, 2019) utilized by the women to solve the challenges presented by our cognitive training contributed to their superior performance in inhibition. Additionally, women tend to experience less decline over time in executive functions compared to men (McCarrey et al., 2016) and it might be that therefore they were more responsive to cognitive training compared to men. It is noteworthy that the women receiving the PTCT only gained additional benefit in inhibition. This might be due to our cognitive training program, which likely fosters goal maintenance, a key requirement for which is inhibition. Hence, the greatest improvement induced by cognitive training was found in the Stroop task (Sipilä et al., 2021).

We found that the additional benefit of the PTCT on inhibition and set- shifting compared to physical training alone was mostly observed in the low-compliance subgroups. In the low-compliance subgroup receiving PTCT, inhibition and set shifting improved by 24 and 7%, respectively, as compared to the 6% improvement in inhibition and the 4% reduction in set shifting in the low-compliance subgroup receiving physical training alone.

Our results showed that different sub-domains of executive functions responded differently to the two interventions, i.e., to the intervention combining PTCT and the intervention comprising physical training alone. It seems that combining PTCT is beneficial enhancing the inhibition of information overload. We found that low-level PTCT benefited inhibition significantly more than low-level physical training alone. A similar trend was observed among participants whose training was on a high level, although the difference between the subgroups was not statistically significant. Set shifting, in turn benefited from low level PTCT, whereas a low level of physical training alone was not sufficient to maintain or improve performance of this task. We therefore suggest that the process underlying the training response in set shifting differs from that underlying inhibition, and hence further investigation is needed to clarify these mechanisms. Our additional subgroup analyses showed (see Supplementary material) some selection bias for compliance and sex. For example, 70% of the men who received PTCT were in the low-compliance subgroup and 30% in the high-compliance subgroup, whereas only 27% of the men receiving physical training alone were in the low- and 73% in the high-compliance subgroup. A similar trend was observed among women. In addition, 70% of the participants who received PTCT and were in the high-compliance subgroup were women and only 30% were men. In the physical training group, the corresponding percentages were 52 and 48%.

Our analyses showed that the training responses in executive functioning were not dependent on age or change in global cognition. To our knowledge, this is the first study to investigate whether these background factors are associated with the greater beneficial effect of the PTCT on executive functions compared to physical training alone. Therefore, more research is needed to investigate the associations of different background factors with intervention-induced improvements in executive functions and determine whether specific subgroups gain additional benefit from the PTCT compared to physical training alone.

The strengths of this study include the representative sample of community-dwelling older adults who did not meet the physical activity guidelines. We used measures appropriate for assessing older adult’s executive functions and global cognition. In addition, the fact that the cognitive measurements were conducted by same research assistant at both time points is likely to enhance the reliability of our results. This study also has its limitations. The main limitation of this study is its hypothesis-generating, exploratory design, which makes it impossible to apply the power calculations of the PASSWORD-study to the subgroup analysis. Moreover, our results cannot be generalized to other age groups or persons with an active lifestyle, adverse medical conditions, or impaired cognitive functions.

In conclusion, PTCT improved older adults’ executive functions, especially among the women and the participants who only occasionally complied with the training regimen. However, the additional benefit from PTCT was uniquely expressed in each of the executive functions investigated in this study. Information provided by this study is of value when developing effective interventions to promote executive functions in older adults.

## Data availability statement

The datasets used during the current study are available from the corresponding author on reasonable request.

## Ethics statement

The studies involving human participants were reviewed and approved by the Ethical Committee of Central Finland Health Care District. The patients/participants provided their written informed consent to participate in this study.

## Author contributions

AT, TT, and SS: acquisition, analysis, interpretation of data, and drafting of the manuscript. TT: statistical analyses. All authors: critical revision of the manuscript, concept, and design.

## Footnotes

1
http://www.isrctn.com/ISRCTN52388040

2
http://www.hur.fi/en

## References

[B1] Barha C. K. Davis J. C. Falck R. S. Nagamatsu L. S. Liu-Ambrose T. (2017). Sex differences in exercise efficacy to improve cognition: A systematic review and meta-analysis of randomized controlled trials in older humans. *Front. Neuroendocrinol.* 46:71–85. 10.1016/j.yfrne.2017.04.002 28442274

[B2] Chandler M. J. Lacritz L. H. Hynan L. S. Barnard H. D. Allen G. Deschner M. (2005). A total score for the CERAD neuropsychological battery. *Neurology* 65 102–106. 10.1212/01.wnl.0000167607.63000.38 16009893

[B3] Diamond A. (2013). Executive functions. *Annu. Rev. Psychol.* 64 135–168. 10.1146/annurev-psych-113011-143750 23020641 PMC4084861

[B4] Fielding R. A. Rejeski W. J. Blair S. Church T. Espeland M. A. Gill T. M. (2011). The lifestyle interventions and independence for elders study: Design and methods. *J. Gerontol. Biol. Sci. Med. Sci.* 66 1226–1237. 10.1093/gerona/glr123 21825283 PMC3193523

[B5] Graf P. Uttl B. Tuokko H. (1995). Color- and picture-word Stroop tests: Performance changes in old age. *J. Clin. Exp. Neuropsychol.* 17 390–415. 10.1080/01688639508405132 7650102

[B6] Grissom N. M. Reyes T. M. (2019). Let’s call the whole thing off: Evaluating gender and sex differences in executive function. *Neuropsychopharmacol. Off. Publ. Am. Coll. Neuropsychopharmacol.* 44 86–96. 10.1038/s41386-018-0179-5 30143781 PMC6235899

[B7] Han K. Tang Z. Bai Z. Su W. Zhang H. (2022). Effects of combined cognitive and physical intervention on enhancing cognition in older adults with and without mild cognitive impairment: A systematic review and meta-analysis. *Front. Aging Neurosci.* 14:878025. 10.3389/fnagi.2022.878025 35928994 PMC9343961

[B8] Harada C. N. Natelson Love M. C. Triebel K. L. (2013). Normal cognitive aging. *Clin. Geriatr. Med.* 29 737–752. 10.1016/j.cger.2013.07.002 24094294 PMC4015335

[B9] Hirvensalo M. Rantanen T. Heikkinen E. (2000). Mobility difficulties and physical activity as predictors of mortality and loss of independence in the community-living older population. *J. Am. Geriatr. Soc.* 48 493–498. 10.1111/j.1532-5415.2000.tb04994.x 10811541

[B10] Karr J. E. Areshenkoff C. N. Rast P. Garcia-Barrera M. A. (2014). An empirical comparison of the therapeutic benefits of physical exercise and cognitive training on the executive functions of older adults: A meta-analysis of controlled trials. *Neuropsychology* 28 829–845. 10.1037/neu0000101 24933486

[B11] Koivisto K. Helkala E. L. Reinikainen K. J. Hanninen T. Mykkanen L. Laakso M. (1992). Population-based dementia screening program in Kuopio: The effect of education, age, and sex on brief neuropsychological tests. *J. Geriatr. Psychiatry Neurol.* 5 162–171. 10.1177/002383099200500306 1497793

[B12] McCarrey A. C. An Y. Kitner-Triolo M. H. Ferrucci L. Resnick S. M. (2016). Sex differences in cognitive trajectories in clinically normal older adults. *Psychol. Aging* 31 166–175. 10.1037/pag0000070 26796792 PMC4783196

[B13] Miyake A. Friedman N. P. Emerson M. J. Witzki A. H. Howerter A. Wager T. D. (2000). The unity and diversity of executive functions and their contributions to complex “Frontal Lobe” tasks: A latent variable analysis. *Cognit. Psychol.* 41 49–100. 10.1006/cogp.1999.0734 10945922

[B14] Muthen L. K. Muthén B. O. (2017). *Mplus User’s Guide*, Eighth Edn. Los Angeles, CA: Muthén & Muthén.

[B15] Paajanen T. Hanninen T. Tunnard C. Mecocci P. Sobow T. Tsolaki M. (2010). CERAD neuropsychological battery total score in multinational mild cognitive impairment and control populations: The AddNeuroMed study. *J. Alzheimers Dis.* 22 1089–1097. 10.3233/JAD-2010-100459 20930314

[B16] Portegijs E. Kallinen M. Rantanen T. Heinonen A. Sihvonen S. Alen M. (2008). Effects of resistance training on lower-extremity impairments in older people with hip fracture. *Arch. Phys. Med. Rehabil.* 89 1667–1674. 10.1016/j.apmr.2008.01.026 18760151

[B17] Reitan R. M. (1958). Validity of the Trail Making Test as an Indicator of Organic Brain Damage. *Percept. Mot. Skills* 8 271–276. 10.2466/PMS.8.7.271-276

[B18] Roig-Coll F. Castells-Sánchez A. Lamonja-Vicente N. Torán-Monserrat P. Pera G. García-Molina A. (2020). Effects of aerobic exercise, cognitive and combined training on cognition in physically inactive healthy late-middle-aged adults: The projecte moviment randomized controlled trial. *Front. Aging Neurosci.* 12:590168. 10.3389/fnagi.2020.590168 33192485 PMC7664521

[B19] Shao K. Wang W. Guo S. Z. Dong F. M. Yang Y. M. Zhao Z. M. (2020). Assessing executive function following the early stage of mild Ischemic stroke with three brief screening tests. *J. Stroke Cerebrovasc. Dis. Off. J. Natl. Stroke Assoc.* 29:104960. 10.1016/j.jstrokecerebrovasdis.2020.104960 32689609

[B20] Sihvonen S. E. Sipila S. Era P. A. (2004). Changes in postural balance in frail elderly women during a 4-week visual feedback training: A randomized controlled trial. *Gerontology* 50 87–95. 10.1159/000075559 14963375

[B21] Sipilä S. Tirkkonen A. Hänninen T. Laukkanen P. Alen M. Fielding R. A. (2018). Promoting safe walking among older people: The effects of a physical and cognitive training intervention vs. physical training alone on mobility and falls among older community-dwelling men and women (the PASSWORD study): Design and methods of a randomized controlled trial. *BMC Geriatr.* 18:215. 10.1186/s12877-018-0906-0 30219032 PMC6139154

[B22] Sipilä S. Tirkkonen A. Savikangas T. Hänninen T. Laukkanen P. Alen M. (2021). Effects of physical and cognitive training on gait speed and cognition in older adults: A randomized controlled trial. *Scand. J. Med. Sci. Sports* 31 1518–1533. 10.1111/sms.13960 33772877

[B23] Ten Brinke L. F. Best J. R. Chan J. L. C. Ghag C. Erickson K. I. Handy T. C. (2020). The effects of computerized cognitive training with and without physical exercise on cognitive function in older adults: An 8-week randomized controlled trial. *J. Gerontol. Biol. Sci. Med. Sci.* 75 755–763. 10.1093/gerona/glz115 31054254

[B24] Zhu X. Yin S. Lang M. He R. Li J. (2016). The more the better? A meta-analysis on effects of combined cognitive and physical intervention on cognition in healthy older adults. *Ageing Res. Rev.* 31 67–79. 10.1016/j.arr.2016.07.003 27423932

