# Supplementary material for: Participant characteristics associated with the effects of a physical and cognitive training program on executive functions
Source: Front Aging Neurosci. 2022 Oct 25;14:1038673. doi: 10.3389/fnagi.2022.1038673 (PMC9640753; doi:10.3389/fnagi.2022.1038673)
Supplement: Supplementary file 1 [file Data_Sheet_1.docx]

**Supplementary materials**

**LONGITUDINAL MODEL**

The outcome variables (Stroop, TMT-B and verbal fluency) were analysed using a path model accounting for the longitudinal relationships among the predictors. For binary predictor variables (sex and compliance) we constructed a four-group model, where within each predictor-intervention group combination (index for predictor *p = 1, 2* and intervention group *g* = 1, PTCT, 2, PT) the two outcome time points for participant *i* (*i* = 1, …, *n_p_*_,_*_g_*) at time point *t* (*t* = 0, 1) were modelled as:

$y_{t,i} = \mu_{t} +\epsilon_{t,i}$, (1)

where $\mu_{t}$is the within-group mean for time point *t*, and the two residual terms were assumed to follow the normal distribution with zero mean vector and covariance matrix:

$\left[ \begin{matrix} \sigma_{0}^{2} & \sigma_{0,1} \\ \sigma_{0,1} & \sigma_{1}^{2} \end{matrix} \right]$,

where $\sigma_{0}^{2}$ is the baseline residual variance, $\sigma_{1}^{2}$ the follow-up residual variance, and $\sigma_{0,1}$ is the covariance between the residuals.

We considered a two-group model for the continuous predictor variable *X* (age and Cerad) within the intervention groups (*g*) for participant *i* (*i* = 1, …, *n_g_*) at time point *t* (*t* = 0, 1) for the assessment of association between the predictor variable *X* and outcome *Y*. The model is given by:

$y_{t,i} = \mu_{t} +\beta_{t}x_{t,i}+\epsilon_{t,i}$, (2)

where the other quantities are defined as in equation (1), $\beta_{t}$ is the time-point-specific effect of predictor *X* on *Y*, and *x_t_*_,_*_i_* is the time-varying value of predictor *X*. It is a notable advantage of the model that it can deal simultaneously with both time-varying covariate values as well as time-dependent effects and model the longitudinal correlation of the outcome measurements.

These models yield the estimated marginal means / regression coefficients that can be used in comparisons based on pre-specified contrasts. Parameters were estimated using the full-information likelihood, enabling flexible treatment of missing values in either or both time points for the outcome. Estimation was performed in Mplus, version 7.4.

**CONTRASTS**

Comparisons for the models shown in equation (1) were based on difference contrasts constructed for maximum likelihood-based estimates of marginal means, $\hat{\mu}$. The following contrasts were used:

1. Changes within predictor (*p*) and intervention (*g*) groups over time (*t*) are shown with the following structure:

$\hat{\mu}_{p,g,.}=\hat{\mu}_{p,g,t=1}-\hat{\mu}_{p,g,t=0}$, (3)

where the dot index refers to difference over groups. This group-structure implies the following differences in our data:

$\hat{\mu}_{1,1,.}=\hat{\mu}_{1,1,1}-\hat{\mu}_{1,1,0}$

$\hat{\mu}_{1,2,.}=\hat{\mu}_{1,2,1}-\hat{\mu}_{1,2,0}$

$\hat{\mu}_{2,1,.}=\hat{\mu}_{2,1,1}-\hat{\mu}_{2,1,0}$

$\hat{\mu}_{2,2,.}=\hat{\mu}_{2,2,1}-\hat{\mu}_{2,2,0}$.

1. Within-predictor comparison of intervention group changes over time had the following structure:

$\hat{\mu}_{p,.,.}=\hat{\mu}_{p,g=1,.}-\hat{\mu}_{p,g=2,.}$. (4)

This structure leads to the following interaction contrasts:

$\hat{\mu}_{1,.,.}=\hat{\mu}_{1,1,1}-\hat{\mu}_{1,1,0}-\hat{\mu}_{1,2,1}+\hat{\mu}_{1,2,0}$

$\hat{\mu}_{2,.,.}=\hat{\mu}_{2,1,1}-\hat{\mu}_{2,1,0}-\hat{\mu}_{2,2,1}+\hat{\mu}_{2,2,0}$.

1. Between-predictor-group comparison of intervention group changes over time had the following structure:

$\hat{\mu}_{.,.,.}=\hat{\mu}_{1,.,.}-\hat{\mu}_{2,.,.}$ (5)

leading to the following interaction contrast:

$\hat{\mu}_{.,.,.}=\hat{\mu}_{1,1,1}-\hat{\mu}_{1,1,0}-\hat{\mu}_{1,2,1}+\hat{\mu}_{1,2,0}-\hat{\mu}_{2,1,1}+\hat{\mu}_{2,1,0}+\hat{\mu}_{2,2,1}-\hat{\mu}_{2,2,0}$.

Comparisons for the models shown in equation (2) were based on contrasts for differences in maximum likelihood-based regression coefficient estimates, $\hat{\beta}$. The following contrasts were used:

1. Changes within intervention (*g*) groups over time are shown using the following structure:

$\hat{\beta}_{g,.}=\hat{\beta}_{g,t=1}-\hat{\beta}_{g,t=0}$. (6)

This contrast structure consists of the following differences:

$\hat{\beta}_{1,.}=\hat{\beta}_{1,1}-\hat{\beta}_{1,0}$

$\hat{\beta}_{2,.}=\hat{\beta}_{2,1}-\hat{\beta}_{2,0}$.

1. Between-intervention group difference in changes over time had the following structure:

$\hat{\beta}_{.,.}=\hat{\beta}_{1,.}-\hat{\beta}_{2,.}$. (7)

which, after plugging in the quantities from equation 6, can be written as:

$\hat{\beta}_{.,.}=\hat{\beta}_{1,1}-\hat{\beta}_{1,0}-\hat{\beta}_{2,1}+\hat{\beta}_{2,0}$.

The contrast defined in this way are analogous to similar quantities that can be computed from e.g., linear mixed models or generalized estimating equations approaches. The main advantages in the path model approach, however, are the more efficient control of missing values and flexibility in the specification of the follow-up correlation structure.

Supplementary Table 1. Participant’s characteristics among women and men. Means and standard deviations. Frequencies and percentages.

|  | WOMEN | | |  | MEN | | |
| --- | --- | --- | --- | --- | --- | --- | --- |
|  | PTCT (N=96) | PT (N=92) | p |  | PTCT (N=59) | PT (N=67) | p |
| Age (years) | 74.5±3.8 | 74.6±3.7 | 0.756^1^ |  | 74.3±4.0 | 74.5±3.8 | 0.609^1^ |
| BMI | 28.2±5.4 | 27.7±5.3 | 0.695^1^ |  | 27.7±4.0 | 28.1±3.2 | 0.235^1^ |
| MMSE (score) | 27.9±1.5 | 27.6±1.5 | 0.168^1^ |  | 27.8±1.2 | 27.2±1.5 | 0.023^1^ |
| CERAD (score) | 79.3±8.3 | 80.9±7.8^7^ | 0.172^2^ |  | 79.9±7.4 | 76.0±8.0 | 0.006^2^ |
| Compliance no (%) |  |  | 0.033^3^ |  |  |  | ˂0.001^3^ |
| Low | 54 (56) | 37(41)^7^ |  |  | 41 (69) | 18 (27) |  |
| High | 42(44) | 54 (59) |  |  | 18 (31) | 49(73) |  |
| Education no (%) |  |  | 0.597 |  |  |  | 0.295^3^ |
| Low | 12 (13) | 9 (10) |  |  | 11 (19) | 16 (24) |  |
| Medium | 59 (61) | 63 (69) |  |  | 35 (59) | 43 (64) |  |
| High | 25 (26) | 20(21) |  |  | 13 (22) | 8 (12) |  |
| Current physical activity no (%) |  | | 0.033 |  |  | | 0.727^3^ |
| Low | 29 (30) | 44 (48) |  |  | 27 (46) | 26 (39) |  |
| Medium | 57 (59) | 38 (41) |  |  | 23 (39) | 30 (45) |  |
| High | 10 (10) | 10 (11) |  |  | 9 (15) | 11 (16) |  |
| Smoking status no (%) |  |  | 0.796 |  |  |  |  |
| Never | 67 (70) | 68 (74) |  |  | 27 (46) | 29 (43) | 0.387^3^ |
| Former | 26 (27) | 22 (24) |  |  | 26 (44) | 35 (52) |  |
| Current | 3 (3) | 2 (2) |  |  | 6 (10) | 3 (5) |  |
| Self-rated health no (%) |  |  | 0.362 |  |  |  | 0.912^3^ |
| very good/good | 46 (48) | 38 (41) |  |  | 27 (46) | 30 (45) |  |
| average/ poor | 50 (52) | 54 (59) |  |  | 32 (54) | 37 (55) |  |

BMI=Body Mass Index, MMSE=Mini-Mental State Examination, BDNF=Brain derived neurotrophic factor, CERAD=The Consortium to Establish a Registry for Alzheimer’s Disease. ^1^Mann-Whitney U-test, ^2^Independent samples t-tets, ^3^Chi-square tes, ^4^n=90, ^5^n=84, ^6^n=86, ^7^n=91, ^8^n=53, ^9^n=64, ^10^n=52, ^11^n=57.

Supplementary Table 2. Participant’s characteristics among compliance groups. Means and standard deviations. Frequencies and percentages.

|  | LOW COMPLIANCE | | |  | HIGH COMPLIANCE | | |
| --- | --- | --- | --- | --- | --- | --- | --- |
|  | PTCT  (n=95) | PT  (n=55) | p |  | PTCT  (n=60) | PT  (n=103) | p |
| Age (years) | 74.7±4.0 | 75.5±3.8 | 0.145^1^ |  | 74.0±3.6 | 74.1±3.7 | 0.949^1^ |
| BMI | 28.0±4.6 | 27.9±4.0 | 0.768^1^ |  | 28.0±5.5 | 27.9±4.8 | 0.594^1^ |
| MMSE (score) | 27.7±1.5 | 27.4±1.5 | 0.154^1^ |  | 28.1±1.4 | 27.4±1.5 | 0.013^1^ |
| CERAD (score) | 78.2±8.2 | 79.0±8.0 | 0.537^2^ |  | 81.6±7.2 | 78.7±8.4 | 0.025^2^ |
| SEX no (%) |  |  | 0.208^3^ |  |  |  | 0.028^3^ |
| Women | 54 (57) | 37 (67) |  |  | 42 (70) | 54 (52) |  |
| Men | 41 (43) | 18 (33) |  |  | 18 (30) | 49 (48) |  |
| Education no (%) |  |  | 0.473^3^ |  |  |  | 0.039^3^ |
| Low | 17 (18) | 13 (24) |  |  | 6 (10) | 12 (12) |  |
| Medium | 58 (61) | 28 (51) |  |  | 36 (60) | 77 (75) |  |
| High | 20 (21) | 14 (25) |  |  | 18 (30) | 14 (14) |  |
| Current physical activity no (%) |  | | 0.990^3^ |  |  | | 0.097^3^ |
| Low | 39 (41) | 23 (42) |  |  | 17 (28) | 46 (45) |  |
| Medium | 46 (48) | 26 (47) |  |  | 34 (57) | 42 (41) |  |
| High | 10 (11) | 6 (11) |  |  | 9 (15) | 15 (15) |  |
| Smoking status no (%) |  |  | 0.129^3^ |  |  |  | 0.575^3^ |
| Never | 60 (63) | 32 (58) |  |  | 34 (57) | 65 (63) |  |
| Former | 27 (28) | 22 (40) |  |  | 25 (42) | 35 (34) |  |
| Current | 8 (8) | 1 (2) |  |  | 1 (2) | 3 (3) |  |
| Self-rated health no (%) |  |  | 0.216^3^ |  |  |  | 0.378^3^ |
| Very good/good | 39 (41) | 17 (31) |  |  | 34 (57) | 51 (50) |  |
| Average/ poor | 56 (59) | 38 (69) |  |  | 26 (43) | 52 (50) |  |

BMI=Body Mass Index, MMSE=Mini-Mental State Examination, BDNF= Brain derived neurotrophic factor, CERAD= The Consortium to Establish for Alzheimer’s Disease. ^1^Mann-Whitney U-test, ^2^Independent samples t-tets, ^3^Chi-square test, ^4^n=87, ^5^n=47, ^6^n=86, ^7^n=46, ^8^n=56, ^9^n=52, ^10^n=101, ^11^n=95.
